# Severe depression is associated with increased microglial quinolinic acid in subregions of the anterior cingulate gyrus: Evidence for an immune-modulated glutamatergic neurotransmission?

**DOI:** 10.1186/1742-2094-8-94

**Published:** 2011-08-10

**Authors:** Johann Steiner, Martin Walter, Tomasz Gos, Gilles J Guillemin, Hans-Gert Bernstein, Zoltán Sarnyai, Christian Mawrin, Ralf Brisch, Hendrik Bielau, Louise Meyer zu Schwabedissen, Bernhard Bogerts, Aye-Mu Myint

**Affiliations:** 1 Department of Psychiatry, University of Magdeburg, Magdeburg, Germany; 2 Pembroke College, University of Cambridge, Cambridge, UK; 3 Institute of Forensic Medicine, Medical University of Gdańsk, Gdańsk, Poland; 4 Department of Pharmacology, University of New South Wales, Sydney, Australia; 5 Department of Pharmacology, University of Cambridge, Cambridge, UK; 6 Institute of Neuropathology, University of Magdeburg, Magdeburg, Germany; 7 Department of Psychiatry, University of Munich, Munich, Germany

## Abstract

**Background:**

Immune dysfunction, including monocytosis and increased blood levels of interleukin-1, interleukin-6 and tumour necrosis factor α has been observed during acute episodes of major depression. These peripheral immune processes may be accompanied by microglial activation in subregions of the anterior cingulate cortex where depression-associated alterations of glutamatergic neurotransmission have been described.

**Methods:**

Microglial immunoreactivity of the N-methyl-D-aspartate (NMDA) glutamate receptor agonist quinolinic acid (QUIN) in the subgenual anterior cingulate cortex (sACC), anterior midcingulate cortex (aMCC) and pregenual anterior cingulate cortex (pACC) of 12 acutely depressed suicidal patients (major depressive disorder/MDD, n = 7; bipolar disorder/BD, n = 5) was analyzed using immunohistochemistry and compared with its expression in 10 healthy control subjects.

**Results:**

Depressed patients had a significantly increased density of QUIN-positive cells in the sACC (*P *= 0.003) and the aMCC (*P *= 0.015) compared to controls. In contrast, counts of QUIN-positive cells in the pACC did not differ between the groups (*P *= 0.558). Post-hoc tests showed that significant findings were attributed to MDD and were absent in BD.

**Conclusions:**

These results add a novel link to the immune hypothesis of depression by providing evidence for an upregulation of microglial QUIN in brain regions known to be responsive to infusion of NMDA antagonists such as ketamine. Further work in this area could lead to a greater understanding of the pathophysiology of depressive disorders and pave the way for novel NMDA receptor therapies or immune-modulating strategies.

## Background

Recent studies have focused on the role of immune dysfunction in depression, and analogies to "cytokine-induced sickness behavior" have been established [1]. Sickness behavior is a coordinated set of adaptive behavioral changes that develop in affected individuals during the course of an infection. Disease symptoms include lethargy, depression, failure to concentrate, anorexia, sleep disturbances, reduction in personal hygiene or social withdrawal, and are mediated by proinflammatory cytokines, such as interleukin-1 (IL-1), interleukin-6 (IL-6) and tumor necrosis factor α (TNFα) [1].

Previous research has suggested that these specific monocyte-derived cytokines are increased in the peripheral blood of acutely depressed patients [2-7] along with elevated monocyte counts [8,9]. Furthermore, lymphocyte and natural killer cell abnormalities have been described [10-12]. It is not yet clear, whether these changes in the peripheral blood are associated with corresponding neuroinflammatory responses and alterations in neurotransmission. Peripheral immune processes may be mirrored in the brains of patients with acute depression by microglial cells which represent the brain's mononuclear phagocyte system (MPS) [2,13]. Indeed, an increased density of microglia expressing human leukocyte antigen (HLA)-DR has recently been observed in the anterior midcingulate cortex (aMCC), the dorsolateral prefrontal cortex and the mediodorsal thalamus of suicidal patients with affective disorders [14]. However, this study of the surface marker HLA-DR did not suggest a mechanism of how modulation of neurotransmission is accomplished.

**Figure 1:**
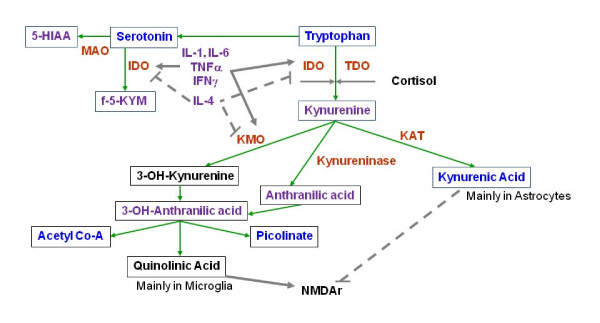
**modified from **[13]**: Tryptophan is an essential amino acid and a precursor for the synthesis of serotonin**. Alternatively, tryptophan can be metabolized in glial cells via the kynurenine pathway to create kynurenic acid (synthesized by kynurenine aminotransferase, KAT) or quinolinic acid (QUIN). These substances are endogenous modulators of NMDA glutamate receptors. A key enzyme of the kynurenine pathway, indoleamine 2,3-dioxygenase (IDO), and the enzyme that catalyses the production of 3-OH-kynurenine, kynurenine monoxygenase (KMO), are activated by proinflammatory cytokines, including interleukin-1 and -6 (IL-1, IL-6), tumor necrosis factor a (TNFa), or interferon g (IFNg). These enzymes are inhibited by anti-inflammatory cytokines, including IL-4. Serotonin is normally broken down into 5-hydroxyindoleacetic acid (5-HIAA), but the indole ring of serotonin can also be cleaved by IDO to form formyl-5-hydroxykynurenamine (f-5-KYM). *Annotation: *grey arrows: activation; dotted grey lines with bar at the end: inhibition; black font: potentially neurotoxic; purple font: neutral or not known; bright blue: potentially neuroprotective.

Quinolinic acid (QUIN), an endogenous modulator with agonistic properties on N-methyl-D-aspartate (NMDA), which is produced by microglial cells, may serve as a potential candidate for such a link between immune and neurotransmitter changes in depression [13]. This hypothesis is based on the observation that the above mentioned proinflammatory cytokines induce a shift from serotonin synthesis to tryptophan metabolism via the kynurenine pathway in glial cells [1,15-17], which may ultimately lead to serotonin depletion and particularly an increased production of the metabolite QUIN (Figure 1). MPS cells, such as microglia, macrophages and monocytes, mainly produce the NMDA receptor agonist QUIN, while astrocytes synthesize the NMDA receptor antagonist kynurenic acid (KYNA) because they lack the enzyme kynurenine monoxygenase (KMO) [18-20]. Analyses of blood and cerebrospinal fluid revealed elevated QUIN levels in cytokine-induced depression and major depressive disorder (MDD) [1,21,22], while an increase in KYNA production was related to schizophrenia [23-25].

These findings may connect immune pathologies to MPS activation in MDD. In addition to serotonin depletion, a direct glutamatergic mechanism has been suggested, which has recently been identified as an important target of antidepressant treatment [26]. In this context, the anterior cingulate cortex (ACC), with its region-specific NMDA and α-amino-3-hydroxy-5-methyl-4-isoxazolepropionic acid (AMPA) glutamate receptor profiles that cover functionally segregated areas, represents an important target region in the central nervous system, although investigations must account for the histo-architectural diversity of this region [27]. The importance of the pregenual anterior cingulate cortex (pACC) in MDD is supported by the pronounced effects of the glutamate modulating NMDA antagonist ketamine on the improvement of clinical symptoms in treatment-resistant MDD patients [28], in which ketamine leads to an increase in glutamate concentration precisely in this region [29].

Therefore, we hypothesized that brain region-specific QUIN synthesis increases in depression and investigated this idea by analyzing the cellular and regional focus of QUIN immunoreactivity in the ACC of depressed suicidal MDD and bipolar disorder (BD) patients. An upregulated production of QUIN by microglia in regions with specific susceptibility to abnormal NMDA throughput would support the hypothesis of an upregulated MPS, and would close the gap between neurochemical imbalances and regional as well as functional *in vivo *imaging findings in depression. Only acutely ill patients were selected for the study, as previous studies of peripheral blood indicate that MPS activation and kynurenine pathway imbalances are associated with acute disease phases. In a postmortem study of chronically stable patients with MDD or BD, transient microglial changes may be missed.

## Methods

### Human brain tissue

Postmortem brains were obtained from the Magdeburg brain bank in accordance with the Declaration of Helsinki and the local institutional review board. Written consent was obtained from the next of kin. The donors were acutely depressed patients (n = 12) who had committed suicide (mean age 51 years; 6 males, 6 females) and controls (n = 10) with no neuropsychiatric illness (mean age 56 years; 5 males, 5 females). The cases were matched with respect to age, gender, duration of disease and autolysis time (Table 1). Patients had been diagnosed with either major depressive disorder (MDD; n = 7) or bipolar disorder (BD; n = 5).

**Table 1:** Demographic data of patients with depression (n = 12) and healthy control subjects (n = 10)

**Case No**.	Diagnosis (DSM-IV)	Gender	Age (y)	Autolysis time (h)	Cause of death
1	Depression, MDD	F	53	47	Suicide by electrocution
2	Depression, MDD	F	46	48	Suicide by hanging
3	Depression, MDD	F	53	46	Suicide by hanging
4	Depression, MDD	F	60	24	Suicide by hanging
5	Depression, MDD	F	68	78	Suicide by intoxication
6	Depression, MDD	M	35	15	Suicide by wrist cutting
7	Depression, MDD	M	36	42	Suicide by hanging
8	Depression, BD	F	46	4	Suicide by intoxication
9	Depression, BD	M	47	24	Suicide by wrist cutting
10	Depression, BD	M	57	48	Suicide by strangulation
11	Depression, BD	M	60	24	Suicide by hanging
12	Depression, BD	M	53	24	Suicide by hanging
	Depression (ratio/mean ± SD)	6F/6M	51 ± 9	35 ± 24	
	MDD (ratio/mean ± SD)	5F/2M	50 ± 12	45 ± 25	
	BD (ratio/mean ± SD)	1F/4MF	53 ± 6	19 ± 10	
13	Control	F	48	48	Status asthmaticus
14	Control	F	50	72	Ruptured aortic aneurysm
15	Control	F	61	8	Sudden death (reason unknown)
16	Control	F	61	24	Heart failure (coronary heart disease)
17	Control	F	63	24	Myocardial infarction
18	Control	M	56	48	Retroperitoneal haemorrhage
19	Control	M	47	24	Acute respiratory failure (aspiration)
20	Control	M	54	35	Ruptured aortic aneurysm
21	Control	M	63	48	Heart failure (after heart surgery)
22	Control	M	54	24	Pulmonary embolism
	Controls (ratio/mean ± SD)	5F/5M	56 ± 6	35 ± 18	
	Statistic (*P *value)	1.000^a^	0.200^b^	0.954^b^	Control vs. Depression
	Statistic (*P *value)	0.214^a^	0.422^c^	0.272^c^	Control vs. MDD vs. BD

*Abbreviations: *BD bipolar disorder, MDD major depressive disorder, F female, M male, SD standard deviation, ^a^chi-square test, ^b^t-test (Control vs. Depression) and ^c^ANOVA (Control vs. MDD vs. BD).

The information used for clinical diagnoses was obtained by carefully studying the patients' clinical records and by structured interviews with physicians involved in patient treatment and with persons who either lived with or had frequent contact with the subjects before death. The DSM-IV axis I diagnosis of MDD and BD was established in consensus meetings of two psychiatrists (JS and HB) using all available information from interviews and clinical records [30]. Brains with lifetime reports of substance abuse, dementia, neurological illness, severe trauma, or chronic terminal diseases known to affect the brain were excluded. Additionally, neuropathological changes due to neurodegenerative disorders, tumors, inflammatory, vascular, or traumatic processes identified by an experienced neuropathologist (CM) were excluded. The determination of suicide was made by a forensic pathologist (TG) and was verified based on the individual records. As summarized in Table 2 the mean daily doses of psychotropic medication taken by patients during the last 90 lifetime days were established according to the clinical files [31-33].

**Table 2:** Mean daily doses of psychotropic medication taken by patients during the last 90 lifetime days

**Case No**.	Antidepressants (amitriptyline equivalents, mg)	Neuroleptics (chlorpromazine equivalents, mg)	Benzodiazepines (diazepam equivalents, mg)	Carbamazepine (mg)	Lithium (mg)
1	67	0	0	0	0
2	124	109	0	0	0
3	0	0	0	0	0
4	100	400	0	0	0
5	100	50	7.5	0	0
6	0	0	0	0	0
7	0	0	0	0	0
8	133	327	3	0	558
9	20	0	0	0	0
10	n.a.	n.a.	n.a.	n.a.	n.a.
11	0	125	10	0	750
12	150	200	0	200	0

*Annotations: *none of these patients was treated with valproate or lamotrigine; n.a. not available.

Tissue preparation was performed as described previously [14,34]. Briefly, brains were fixed in 8% phosphate-buffered formaldehyde (pH 7.0) for three months. Subsequently, after separation of the brainstem and the cerebellum, the hemispheres were divided by coronal cuts into three bi-hemispherical coronal blocks comprising the frontal lobe anterior to the genu of the corpus callosum ("anterior" block), the fronto-temporo-parietal lobe extending the entire length of the corpus callosum ("middle" block) and the occipital lobe ("posterior" block). After embedding the brains in paraffin, serial coronal whole brain sections were cut 20 μm in width and mounted.

### Region selection

**Figure 2:**
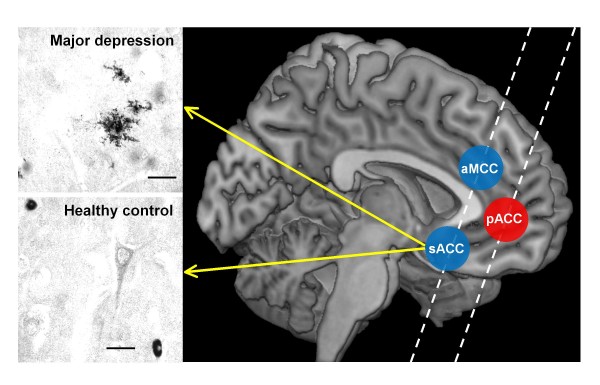
**Illustrations of QUIN-immunoreactive cells from the left sACC of a depressed suicidal patient and a control case and the locations of the analyzed regions of interest (sACC, aMCC and pACC)**. Depressed patients showed microglial formations with numerous granular structure processes. *Annotation: *Scale bars represent 20 μm.

Sections for QUIN immunohistochemistry were anatomically selected corresponding to Brodmann's area (BA) 24' (anterior midcingulate cortex, aMCC), BA 25 (subgenual anterior cingulate cortex, sACC) and BA 24/32 (pregenual anterior cingulate cortex, pACC) for QUIN immunohistochemistry (Figure 2) [27,35]. We were able to study both subgenual and supracallosal areas in the same section. These two regions have similar receptor architectonics, in contrast to a more pregenual region of the ACC, which was covered by a second section. This method was possible given the suitable angulation of the coronal whole brain sections available in the Magdeburg brain bank.

The exact thickness of each section was determined by focusing on the upper and lower surfaces of the section and subtracting the z-axis coordinate of the lower surface from that of the upper surface. The movements in the z-axis were measured with a microcator, part of the Leica DM RB microscope (Leica, Gießen, Germany). The section thickness after histological procedures was 18.7 ± 1.1 μm (mean ± SD).

### Immunohistochemistry

Formalin-fixed tissue sections were deparaffinized, and antigen demasking was performed by boiling the sections for 4 min in 10 mM citrate buffer (pH 6.0). Preincubation with 1.5% H_2_O_2 _for 10 min to block endogenous peroxidase activity was followed by blocking non-specific binding sites with 10% normal goat serum for 60 min and repeated washings with PBS. Next, a polyclonal rabbit QUIN antibody was used (ab37106, Abcam, Cambridge, UK) at a dilution of 1:150 for 72 h at 4°C. Sections were then incubated with a biotinylated goat anti-rabbit secondary antibody (Amersham, Little Chalford, UK) for the streptavidin-biotin technique. Chromogen 3,3'-diaminobenzidine (DAB) and ammonium nickel sulfate were used to visualize the reaction product [36]. The specificity of the polyclonal rabbit primary antibody was confirmed by a loss of signal after preabsorption of 2 ml of the primary antibody solution (dilution 1:150) with 1 mg QUIN (Sigma-Aldrich, Munich, Germany) for 24 h and by the supplier's ELISA competition experiments with QUIN, kynurenic acid and phenylalanine.

### Quantification

Immunopositive cells were counted in the delineated brain regions listed above at 200× magnification (Olympus BH2, Olympus, Hamburg, Germany) by experimenters blind to the donors' diagnoses (TG and LMS). Evaluations were performed in two coronal sections per brain region of interest. The counting area was measured with the graphical analysis software Digitrace v. 2.10a (Imatec, Miesbach, Germany) using a SZX12 stereomicroscope (Olympus, Hamburg, Germany). The cytological classification of immunopositive cells as microglia, astrocytes, oligodendrocytes or neurons was performed according to established cytomorphological criteria [37]. Cells visibly located inside vessels were classified as monocytes; only cells that were clearly outside the vessels and situated in tissue were evaluated. Cell densities were calculated by dividing the cell number by the counting area multiplied by the section thickness [cells/mm^3^].

### Statistical analysis

Statistical analyses were performed with the SPSS 15.0 program (Statistical Product and Service Solutions, Chicago, IL, USA). Demographic data were compared by the chi-square test, t-test and analysis of variance (ANOVA). QUIN data were not normally distributed, as indicated by the Kolmogorov-Smirnov test. Therefore, Spearman's rank correlation coefficient, the Kruskal-Wallis H test and the Mann-Whitney U test were employed. These non-parametric tests were further used to explore potential confounds due to age, gender, duration of disease, method of suicide, autolysis or fixation time, and medication dosage.

## Results

### Qualitative evaluation

Strong QUIN immunoreactivity was found exclusively in vascular monocytes and microglial cells. In contrast, faint staining was only occasionally observed in fibers and other cell types, such as pyramidal neurons and astroglia. The immunoreactive microglia revealed different morphological features in healthy controls versus patients. In control subjects, we found mostly a smooth, ovoid or elongated cell form (Figure 2). In contrast, particularly in the aMCC and the sACC, the cortical grey matter of depressed patients revealed microglial forms with numerous granular structure processes (Figure 2), as previously demonstrated by Guillemin et al. in human tissue [38].

### Quantitative evaluation

**Figure 3:**
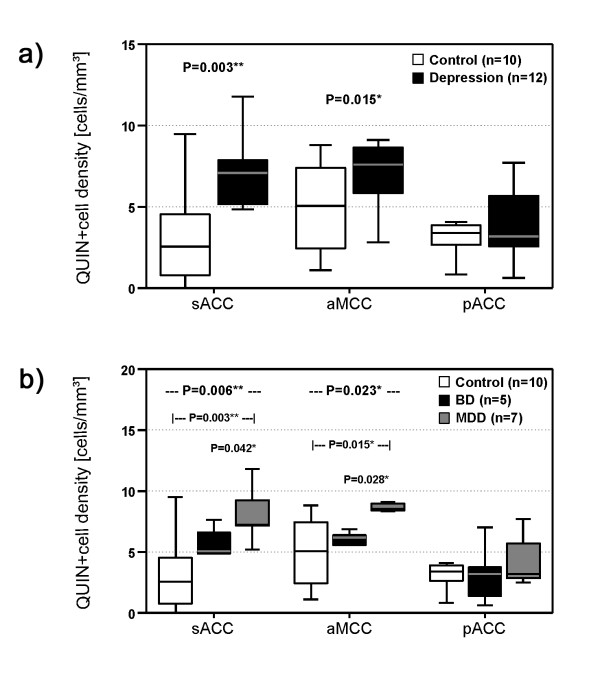
**Illustration of QUIN-immunopositive cell densities.** a) Depressed patients had increased QUIN-immunopositive cell densities in the sACC and the aMCC but not in the pACC. b) MDD patients showed the highest QUIN-immunoreactive cell counts in the sACC and the aMCC compared to BD and control cases. No diagnostic subgroup-dependent differences were observed in the pACC. *Annotation: *The box plots show the median, interquartile range, sample minimum and sample maximum, * *P *< 0.05, ** *P *< 0.01.

Comparing QUIN-immunopositive microglia between depressed patients and healthy controls revealed a region-specific pattern with group effects only in the aMCC and the sACC. Depressed patients had significantly increased QUIN-positive cells in the sACC (*P *= 0.003) and the aMCC (*P *= 0.015). In contrast, cell counts in the pACC did not differ between groups (*P *= 0.558) (Figure 3a).

Post-hoc tests of diagnostic subgroups identified increased cell counts only for MDD patients. In these patients, QUIN-immunopositive microglia was increased compared to controls (sACC *P *= 0.003, aMCC *P *= 0.015) and compared to the subgroup of bipolar depressed cases (sACC *P *= 0.042, aMCC *P *= 0.028) (Figure 3b). Notably, no significant increase was found in the pACC in either comparison. Diagnostic specificity of the increases in MDD was further supported by the lack of any significant increase or decrease in QUIN-immunopositive microglia cell counts in bipolar depressed patients when compared to healthy controls.

The reported effects were controlled for the potential confounding factors of age, gender, duration of disease, method of suicide, autolysis or fixation time, and medication dosage.

## Discussion

To our knowledge, this is the first report of microglial QUIN expression in human brain during acute depressive episodes. An increase in QUIN-immunopositive microglia was specific to cingulate subregions with high NMDA receptor densities, like the sACC and the aMCC, but not the pACC, which shows a lower NMDA receptor expression. This increase in QUIN-immunoreactive microglial cell densities was found particularly in unipolar patients. With regard to BD less clear statements can be given. We observed a significant difference between MDD and BD, yet the BD group is also higher than the controls, though this is apparently not significant (Figure 3b). This could be due to the small number of specimens studied. The numeric increase in QUIN-immunopositive cell counts was paralleled by the presence of microglial forms that displayed numerous granular structure processes in the proximity of neurons in the depressed group, supporting an interaction of inflammatory mechanisms and neurotransmission at the time of acute depressive episodes. These findings thus corroborate evidence for acute inflammatory microglial activation in depression, leading to increased levels of the NMDA receptor agonist QUIN in regions with corresponding receptor profiles that have been previously revealed as key structures in non-invasive imaging studies.

Increased levels of QUIN, which is also produced by macrophages and monocytes, have already been found in the blood and cerebrospinal fluid of subjects with cytokine-induced depression or MDD [1,21,22]. Thus, our result of increased microglial QUIN expression in suicidal MDD patients is in line with the hypothesis of a systemic MPS activation during acute disease phases of depression [2-9,14]. Due to the excitotoxic properties of QUIN, our findings are also supporting the neurodegeneration hypothesis of depression [15]. Therefore, our study provides insight into why immune- and glutamate-modulating therapies may be helpful for acutely ill suicidal patients suffering from depression. Potential candidate drugs include the tetracycline antibiotic minocycline, which inhibits microglial activation by blocking NF-kappa B nuclear translocation [39-42] or anti-inflammatory inhibitors of cyclooxygenase-2 [43,44]. Furthermore, severely depressed suicidal patients may benefit from the administration of glutamate-modulating drugs, such as the NMDA receptor antagonist ketamine [28,45,46].

It should be mentioned that Laugeray and colleagues observed reduced levels of the QUIN precursor 3-OH-kynurenine (3HK) in the cingulate cortex and increased levels of 3HK in the striatum and the amygdala of mice using an unpredictable chronic mild-stress model for the induction of depressive-like symptoms [47]. The observation of reduced 3HK could be due to either reduced formation of 3HK or increased degradation of 3HK to QUIN, which would result in reduced 3HK level. Since QUIN was not directly measured in this study, a translational validation of these converging results remains subject to future studies. A general drawback of animal studies is that it is unclear if animal models adequately reflect the pathophysiology of human MDD or BD. Moreover, an analysis of ACC subregions was not undertaken in this study, and direct correspondence of subregions in primates and humans differ considerably to those found in rodents. Therefore, the implications on regional glutamatergic throughput in depression, as a function of local NMDA and AMPA receptor profiles, remain difficult to interpret in animal studies.

We have shown that abnormal NMDA receptor function related to microglial activation is highly dependent on the location in the ACC in humans. Non-invasive studies have led to similar distinctions of abnormal cingulate cortex activation in MDD. While sACC hyperactivity has been postulated in a number of studies, the pACC has been less consistently characterized. Grimm et al. [48] found a reduced deactivation during a task study, reflected in smaller negative BOLD responses in a sample of severely depressed patients; this functional deficit was accompanied by decreased pACC glutamate and glutamine levels, which are correlated with the severity of clinical depressive symptoms [49-51]. Moreover, these glutamatergic deficits have been related to anhedonia and abnormal functional activations in the pACC in humans [52]. Our finding of relatively increased QUIN immunoreactivity, which is potentially associated with serotonin depletion due to changes in the kynurenine pathway, would thus be consistent with the relative hyperactivation in the sACC. The sACC is also a putative target of deep brain stimulation. Importantly, the metabolic activity after deep brain stimulation in the sACC, as measured by positron emission tomography, shows a reduction in hyperactivity similar to a region bordering the aMCC and the pACC [53].

Specifically increased concentrations of the NMDA receptor agonist QUIN in the aMCC and the sACC may also directly contribute to the disturbed balance in glutamatergic throughput, which could explain the rapid onset of antidepressant effects after ketamine [28,46]. According to Salvadore et al. [54], activity bordering the pACC does indeed predict the responsiveness towards ketamine treatment; therefore, our finding may represent a histopathological surrogate. As shown by Vollenweider and Kometer [55], similar metabolic changes can be found in the sACC and aMCC upon acute ketamine administration. Therefore, the anatomical patterns of such pharmacological challenges fit the observed pattern of microglial histopathology.

The present study has certain limitations that need to be considered: (1) our findings are based on a relatively small number of MDD and BD cases and must be confirmed in a larger sample size; (2) it was not possible to track data on drug exposure or the history of inflammation and infection across the patients' entire life spans, as we could only collect data on psychotropic medication in the three months prior to death; (3) the present study enables us to draw conclusions about the cellular QUIN content, but not released or secreted QUIN in the extracellular space, which potentially interferes with glutamatergic neurotransmission; (4) it remains unclear if increased QUIN immunoreactivity in microglial cells is caused by increased synthesis or reduced degradation of QUIN. Future studies in frozen tissue may address this question by measuring different kynurenine pathway metabolites using high-performance liquid chromatography (HPLC) or mass spectrometry (MS). (5) It is currently uncertain if drugs like glibenclamide, nifedipine, metoprolol, or theophylline which have been applied in five of the control subjects may influence microglial QUIN expression.

## Conclusion

Here we present the first study providing evidence that supports a disease-related upregulation of microglial QUIN in depressive disorders, particularly in brain regions known to be responsive to infusion of NMDA antagonists such as ketamine [55]. These results add a novel link to the immune [1,26] and neurodegeneration [15] hypotheses of depression. Further work in this area could lead to a greater understanding of the pathophysiology of depressive disorders and pave the way for identification of novel biomarkers and therapeutic strategies targeting specific disease subtypes.

## Competing interests

The authors declare that they have no competing interests.

## Authors' contributions

The work presented here has been carried out in collaboration between all authors. JS, MW, TG, GJG, HGB, BB and AMM have designed the study. CM has done the routine neuropathological examination. DSM-IV axis I diagnosis of MDD and BD was established in consensus meetings of JS and HB. JS, TG, HGB and LMS carried out the laboratory experiments. JS, TG, GJG, LMS and AMM analyzed the data and interpreted the results. RB was involved in the creation of figures. JS, MW, TG, ZS, BB and AMM wrote the manuscript. All authors have read and approved the final version of the manuscript.
