# Correction: Severe depression is associated with increased microglial quinolinic acid in subregions of the anterior cingulate gyrus: evidence for an immune-modulated glutamatergic neurotransmission?

**DOI:** 10.1186/1742-2094-10-34

**Published:** 2013-03-02

**Authors:** Johann Steiner, Martin Walter, Tomasz Gos, Gilles J Guillemin, Hans-Gert Bernstein, Zoltán Sarnyai, Christian Mawrin, Ralf Brisch, Hendrik Bielau, Louise M zu Schwabedissen, Bernhard Bogerts, Aye-Mu Myint

**Affiliations:** 1 Department of Psychiatry, University of Magdeburg, Magdeburg, Germany; 2 Pembroke College, University of Cambridge, Cambridge, UK; 3 Institute of Forensic Medicine, Medical University of Gdańsk, Gdańsk, Poland; 4 Department of Pharmacology, University of New South Wales, Sydney, Australia; 5 Department of Pharmacology, University of Cambridge, Cambridge, UK; 6 Institute of Neuropathology, University of Magdeburg, Magdeburg, Germany; 7 Department of Psychiatry, University of Munich, Munich, Germany

## Correction

After publication of the article [1] an error in figure 3 was noticed. The P-values denoted in figure 3a (sACC: P= 0.003 and aMCC: P= 0.015) are in fact the results from the second analysis which are denoted correctly in figure 3b (relating to the diagnostic subgroups MDD versus controls). The accurate corresponding values for figure 3a should be sACC: P= 0.006 and aMCC: P= 0.043. See Figure 1.

**Figure 1:**
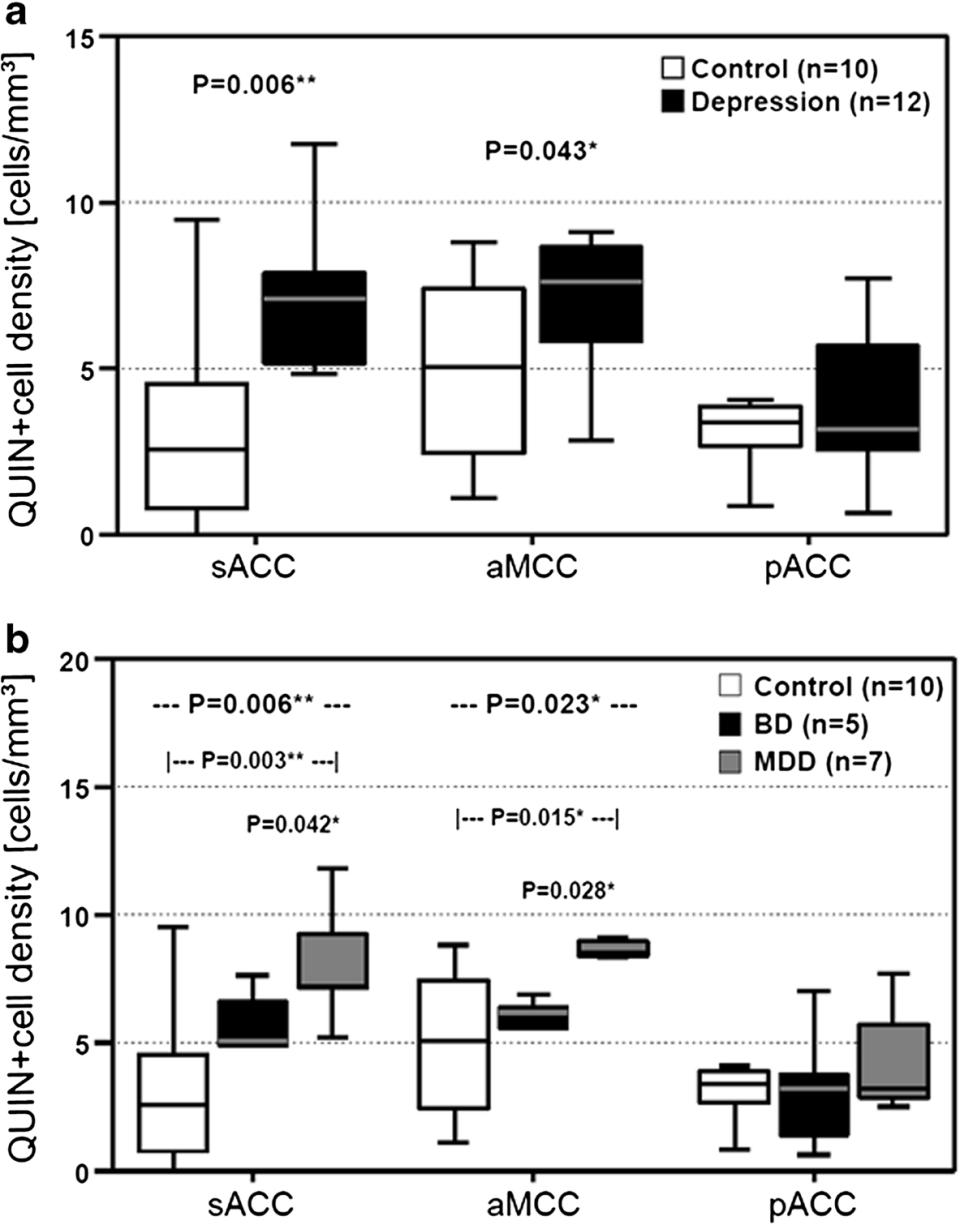
**Illustration of QUIN-immunopositive cell densities. a) **Depressed patients had increased QUIN-immunopositive cell densities in the sACC and the aMCC but not in the pACC. **b) **MDD patients showed the highest QUIN-immunoreactive cell counts in the sACC and the aMCC compared to BD and control cases. No diagnostic subgroup-dependent differences were observed in the pACC. *Annotation: *The box plots show the median, interquartile range, sample minimum and sample maximum, * *P *< 0.05, ** *P *< 0.01.

The interpretation of results and the conclusions drawn are not affected as the level of significance is the same (sACC: P< 0.01 and aMCC: P< 0.05). Accordingly, the proper summary of results in the abstract and results section is:

"Depressed patients had a significantly increased density of QUIN-positive cells in the sACC (P= 0.006) and the aMCC (P= 0.043) compared to controls."

The authors apologize for this inadvertence and any inconvenience caused.
